# Research advances in smart responsive-hydrogel dressings with potential clinical diabetic wound healing properties

**DOI:** 10.1186/s40779-023-00473-9

**Published:** 2023-08-23

**Authors:** Ying Chen, Xing Wang, Sheng Tao, Qi Wang, Pan-Qin Ma, Zi-Biao Li, Yun-Long Wu, Da-Wei Li

**Affiliations:** 1 Fujian Provincial Key Laboratory of Innovative Drug Target Research and State Key Laboratory of Cellular Stress Biology, School of Pharmaceutical Sciences, Xiamen University, Xiamen, 361102 Fujian China; 2 Beijing National Laboratory for Molecular Sciences, Institute of Chemistry, Chinese Academy of Sciences, Beijing, 100090 China; 3 University of Chinese Academy of Sciences, Beijing, 100049 China; 4 Senior Department of Orthopedics, the Fourth Medical Center of PLA General Hospital, Beijing, 100091 China; 5 Institute of Materials Research and Engineering, A*STAR (Agency for Science, Technology and Research), Singapore, 138634 Singapore

**Keywords:** Responsive-hydrogel, Diabetic wound, Anti-inflammation, Tissue remodeling

## Abstract

The treatment of chronic and non-healing wounds in diabetic patients remains a major medical problem. Recent reports have shown that hydrogel wound dressings might be an effective strategy for treating diabetic wounds due to their excellent hydrophilicity, good drug-loading ability and sustained drug release properties. As a typical example, hyaluronic acid dressing (Healoderm) has been demonstrated in clinical trials to improve wound-healing efficiency and healing rates for diabetic foot ulcers. However, the drug release and degradation behavior of clinically-used hydrogel wound dressings cannot be adjusted according to the wound microenvironment. Due to the intricacy of diabetic wounds, antibiotics and other medications are frequently combined with hydrogel dressings in clinical practice, although these medications are easily hindered by the hostile environment. In this case, scientists have created responsive-hydrogel dressings based on the microenvironment features of diabetic wounds (such as high glucose and low pH) or combined with external stimuli (such as light or magnetic field) to achieve controllable drug release, gel degradation, and microenvironment improvements in order to overcome these clinical issues. These responsive-hydrogel dressings are anticipated to play a significant role in diabetic therapeutic wound dressings. Here, we review recent advances on responsive-hydrogel dressings towards diabetic wound healing, with focus on hydrogel structure design, the principle of responsiveness, and the behavior of degradation. Last but not least, the advantages and limitations of these responsive-hydrogels in clinical applications will also be discussed. We hope that this review will contribute to furthering progress on hydrogels as an improved dressing for diabetic wound healing and practical clinical application.

## Background

In 2021, 10% of the population worldwide have diabetes, according to the latest report of the International Diabetes Federation (IDF), by 2030 and 2045, this number is expected to climb to 643 million and 783 million, respectively [1]. As one of the most significant and prevalent chronic diseases, diabetes is frequently associated with chronic wounds [2–4], in terms of diabetic foot ulcer (DFU). In details, the prevalence of DFU is around 15.0% in South East Asia, 10.0–30.0% in Africa, 21.0% in Brazil, 1.0–17.0% in Europe, and 5.0–20.0% in the Middle East or North Africa [5]. More importantly, diabetes patients with chronic wounds, like DFU, might experience a risk of recurrent infection and amputation, which significantly increases morbidity or death worldwide with unaffordable healthcare costs [6]. Not only associated with hemostasis, inflammation, proliferation, and tissue remodeling [7, 8], the environment around a diabetic wound is more complex, leading to overlapped wound healing processes and protracted inflammatory period [9]. As a consequence, diabetic wounds are prone to reoccur, and are often incompletely healed. And even in extreme circumstances, diabetic wounds are prone to result in amputation or even death [9]. In short, diabetic wound healing has become a significant burden for global healthcare systems [4].

Wound dressing plays an important role in the clinical treatment of diabetic wounds [10]. Traditional wound dressings such as gauze bandages can stop bleeding, absorb wound exudates and help to protect the wound and prevent infection, but these dressings do not speed up the healing process [11, 12]. Actually, frequent dressing replacement is more likely to cause secondary or even multiple injuries, which will reduce patient compliance [11, 13]. According to recent moist wound healing theory, the ideal dressing should offer an environment that has adequate humidity, proper temperature, and pH values, and is easy to be removed without harming skin tissue cells [11, 14]. Furthermore, a dressing with excellent hemostasis maintenance capabilities, as well as anti-infection and pro-repair capabilities might be suitable for diabetic wounds. Typically, foam, film, and hydrogel are currently commercially available wet wound dressings [15]. Among these dressings, hydrogels have advantages in terms of biocompatibility, moisture retention, and transparency, which permits visual monitoring of wounds [16, 17]. Hyaluronic acid dressings (Healoderm) [18], Aquaform (Maersk Medical), Intrasite Gel (Smith and Nephew) [19] and other hydrogel wound dressings that can absorb wound exudate, promote autolysis of necrotic tissue and maintain the moist environment of a wound have achieved good clinical wound treatment effect. Nonetheless, the complexity of the diabetic wound microenvironment is reflected in its characteristics of high blood glucose level, high levels of reactive oxygen species (ROS), low pH and abnormal matrix metalloproteinase (MMP) level, all of which increase the risk of infection, poor angiogenesis, and impaired healing [20]. Although hydrogel dressings can be used in combination with antibiotics or other drugs, the current clinical use of hydrogel wound dressings cannot respond to the wound characteristics of diabetes, which can lead to inappropriate drug application, resulting in poor efficacy or drug resistance [21]. These concerns pose new challenges for the application of clinical diabetic wound dressings and point the way for their design.

To overcome the limitations of clinical diabetic wound dressings, smart responsive hydrogels are being developed to respond to specific diabetic wound environments (such as low pH, high ROS, high glucose, and overexpressed enzymes) or external conditions (such as temperature, light, and magnetism) [22–26]. These smart responsive hydrogels can achieve precise and control drug release on demand, avoiding drug resistance caused by high-dose antibiotic use and improving the harsh microenvironment of diabetic wound sites. For example, Wu et al*.* [27] developed pH-responsive hydrogels (DP7-ODex hydrogels) loaded with ceftazidime and antimicrobial peptide DP7. In acidic environments, hydrogel degradation accelerates drug release, while DP7 damages bacterial cell membranes, significantly reducing the inhibitory dose of ceftazidime on multiple drug resistance (MDR) bacteria. Such smart responsive hydrogels designed according to the characteristics of diabetic wounds may be superior to existing medical dressings and show promise in the treatment of diabetic chronic wounds [28].

**Fig. 1:**
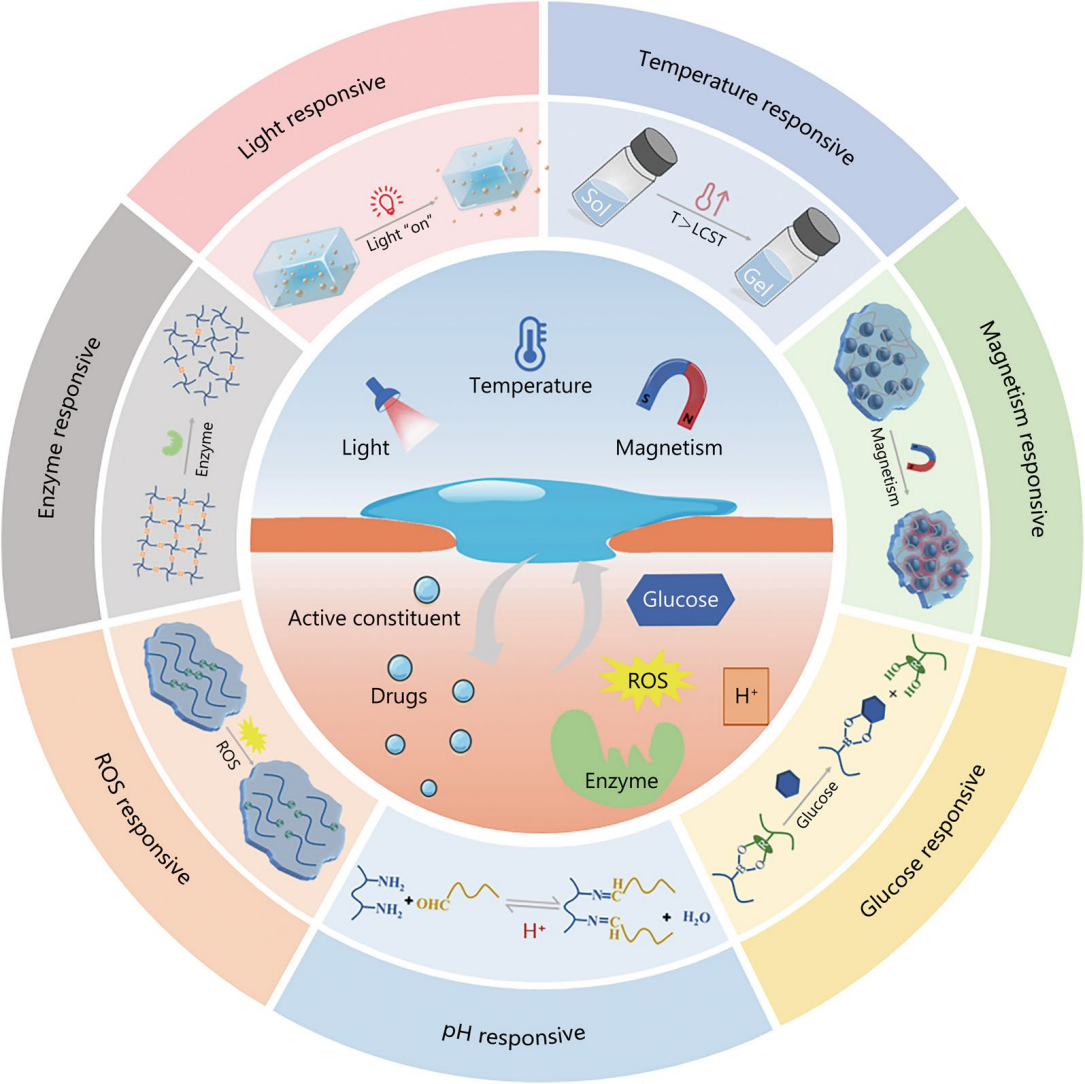
Schematic illustration showing smart responsive hydrogel wound dressings for the treatment of diabetic wounds. ROS reactive oxygen species, T temperature, LCST lower critical solution temperature, Sol solution

In this report, we aim to summarize the advantages and development prospects of existing clinical hydrogel dressings and intelligent responsive hydrogel dressings towards diabetic wound treatment, from the perspective of clinical application. In details, the environment of diabetic wounds and the factors that impede diabetic wound healing will be discussed. Furthermore, the advantages of hydrogels in the treatment of diabetic wounds will be introduced with a focus on the potential application of smart responsive hydrogels in diabetic wound healing (Fig. 1). In short, this review is intended to contribute to the ongoing work to promote hydrogels towards ideal dressing for diabetic wound healing which might be beneficial for clinical application of hydrogels.

## Wound healing process and challenges in diabetic wound healing

### Normal wound healing process

Normal wound healing is a well-organized process divided into four overlapping stages: hemostasis, inflammation, proliferation, and remodeling [14, 16, 29, 30]. During these processes, many cells and components involved work together to rebuild injured skin tissue and restore its integrity [31]. The hemostatic and inflammatory phases are primarily concerned with wound healing and pathogen resistance, respectively, which are performed by the production and action of platelets, neutrophils, and macrophages [32]. In the proliferation phase, secretory factors increase vascular permeability and edema, and endothelial, epidermal, and dermal cells congregate around the wound site as a consequence of growth factor and cytokine release [33]. The proliferation phase progresses to angiogenesis, at which time fibroblasts and collagen synthesis increase, culminating in the formation of granulation tissue [34]. After 1–3 weeks, tissue remodeling occurs through fibroblast differentiation into myoblasts, an increase in type I collagen, and the recovery of the extracellular matrix (ECM) [35, 36]. The freshly formed blood vessel network rapidly progresses into a mature tissue structure with limited structural strength and a reduced number of resident cells, eventually resulting in scar tissue formation [37]. Although, in diseased condition like diabetic, the wound healing process also consists of these four stages, the complex microenvironment can hinder progress and lead to the formation of chronic wounds [38].

### Hindrance to diabetic wound healing

**Fig. 2:**
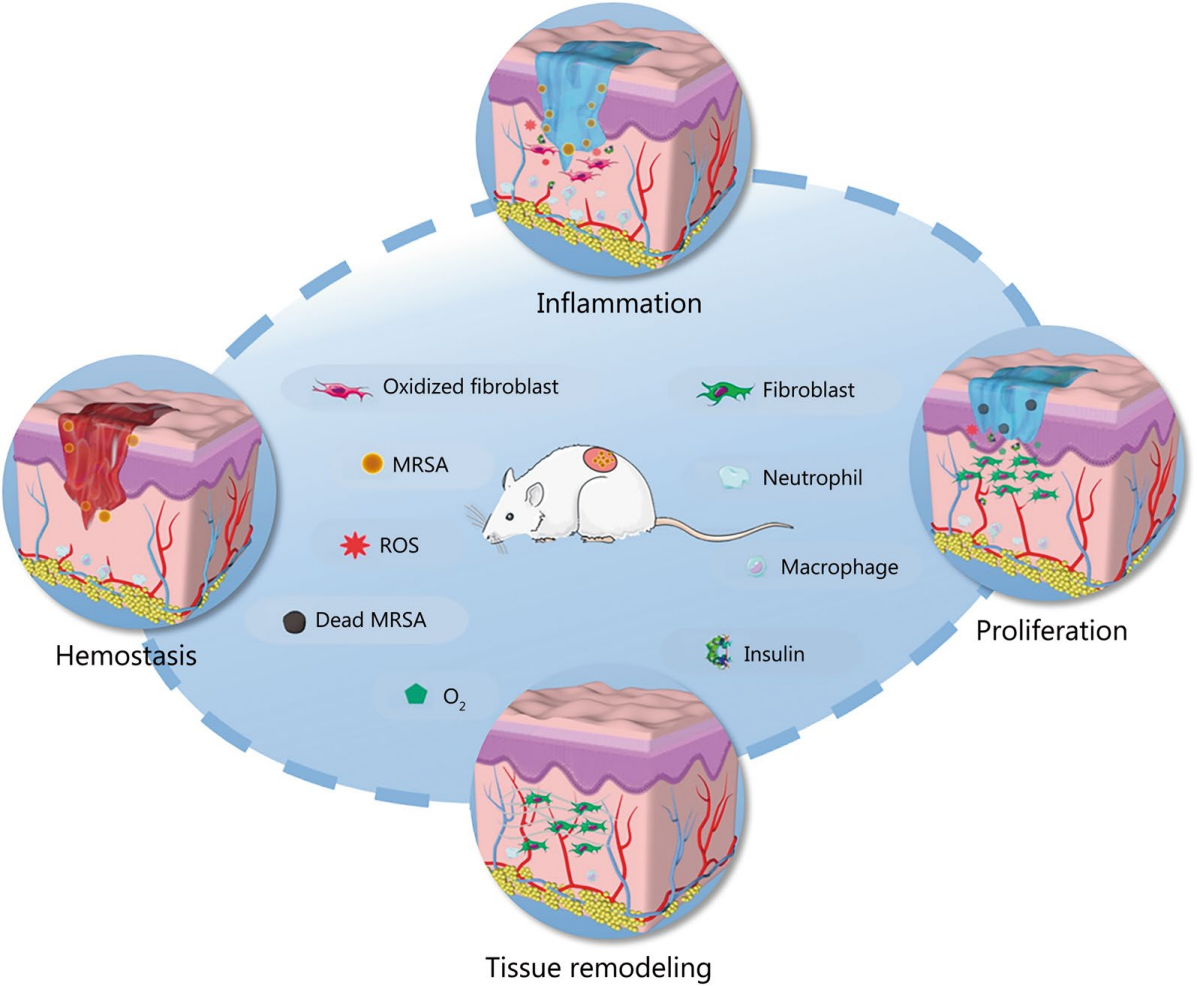
Diabetic wound healing process. Reproduced with permission [44]. Copyright 2020, American Chemical Society. ROS reactive oxygen species, MRSA methicillin-resistant* Staphylococcus aureus*

Compared with typical wounds and other types of chronic wounds, diabetic wounds possess complex microenvironments. For example, DFU, as one of the most serious complications of diabetes, has a complex pathogenesis and is difficult to treat [39]. The main reason is that DFU microenvironment is rather complicated and characterized by hyperglycemia, hypoxia, excessive wound exudates, recurrent bacterial infection, accumulation of ROS, disordered expression of cell factors and growth factors, increased protease activity, persistent inflammation, and impaired angiogenesis and tissue regeneration [40–43]. All these factors can impede the healing process, prolonging one or more of the four overlapping stages of wound healing [44] (Fig. 2) and increasing the difficulty of clinical diabetic wound treatment.

#### High glucose level

Clinically, high blood glucose levels at the site of injury not only lead to cell damage and vascular lesions but also greatly increase the risk of bacterial infection. For example, the formulation of advanced glycosylation end products (AGEs) can directly activate immune cells to produce high levels of ROS that eventually result in elevated oxidative stress, destruction of cell redox balance and aggravation of metabolic disorders in the wound area [45–47]. In addition, AGEs can undermine macrophage transformation from typical activated (M1) macrophages with pro-inflammatory properties to alternately activated (M2) macrophages with anti-inflammatory and tissue repair functions [48]. Excessive M1 macrophage infiltration leads to continuous aggregation and activation of pro-inflammatory cells at the lesion site, resulting in prolonged inflammation [49]. Elevated blood sugar levels can also harden cell membranes and constrict blood vessels, impairing healing by lowering blood flow and depriving the wound site of nutrients and oxygen [50]. Beyond that elevated blood glucose levels can provide increased nutrient resources for bacteria growth and proliferation, leading to recurrent bacterial infection [51]. High blood glucose level, one of the most prominent features of diabetic wound environments, is thus the biggest obstacle to healing. Therefore, in the clinical treatment of diabetic wounds, the first requirement is to control blood sugar levels.

#### Wide and varying pH

The pH value of wounds is dynamic and affected by several variables during the wound-healing process which can influence bacterial infection, enzyme activity, oxygen supply, cell proliferation and other factors. To fight microbial invasion, the pH of the skin surface is normally slightly acidic at 4–6 [52]. When skin is damaged, the underlying tissue with internal pH milieu of 7.4 is exposed, showing weak alkalinity. Wounds typically transition from alkaline to acidic as they heal, but the pH of chronic and infected wounds stays alkaline for an extended time owing to the continuous inflammation. Dissemond et al*.* [53] assessed pH values in 39 patients with chronic wounds from various causes and found pH ranged from 5.45 to 8.65. As in acute wounds, the pH level of the diabetic wound typically starts as alkaline and gradually progresses to neutral and subsequently to an acidic state [50], however, prolonged inflammation often leads to lower pH at the wound bed [26]. The higher initial pH value of the microenvironment of chronic wounds compared to normal skin makes it more conducive to bacterial growth and reproduction, increasing the risk of long-term bacterial infection [54]. Schneider et al*.* [52] summarized a number of clinical studies and showed that chronic and infectious wounds remained in alkaline pH environment. However, most of the currently reported pH-responsive hydrogel wound dressings are designed to be degraded at low pH, probably because of the consideration that bacteria at the diabetic wound site can convert glucose to lactic acid, creating an even lower pH environment [42]. Furthermore, this contradiction might be due to differences between animal models and diabetic patients. The duration of diabetic wounds in experimentally constructed animals is shorter and might still be in the acute phase, when the environmental pH is acidic. This situation is different from the environment (pH > 7.3) in the chronic stage of clinical diabetes patients [52]. Last but not least, the wound pH value depends on time course and wound-stage, such as chronic wounds in the healing process will also show acidic pH. In short, pH at diabetic wound sites varies widely and is related to many factors such as microbial colonization and the stage of wound healing progression. Therefore, the design of wound dressings, with ability to accurately regulate the pH value at the diabetic wound, may significantly promote the wound healing process.

#### Hypoxia

Clinical reports have shown that oxygen is essential in the wound healing process, which has served as a therapeutic approach to aid and speed wound healing. The regulation of energy metabolism, oxidative stress and bacterial drug resistance is important at various stages of wound healing and is affected by oxygen content in the microenvironment [55]. In diabetic trauma, capillary damage and impaired angiogenesis can lead to inadequate oxygen supply and decreased immune response, which in turn exacerbates bacterial infection and local inflammation. In clinical settings, hyperbaric oxygen therapy is used, when necessary, in the treatment of diabetic foot patients to promote wound healing and decrease amputation risk in patients [56, 57]. It is worth mentioning that hyperbaric oxygen therapy is still an expensive therapeutic approach. Therefore, it might also be valuable to design convenient wound dressings, with full consideration of its impact on the wound environment oxygen.

#### High levels of ROS

Normal levels of ROS, which act as a secondary messenger of many immune and non-lymphocyte cells, are effective in promoting angiogenesis and resisting bacterial infection. However, long-term excessive ROS levels can cause chronic inflammation and irreversible cell damage in the microenvironment, making the wound more fragile, and inhibiting the function of endogenous stem cells and macrophages, which hinders wound healing [58]. In diabetic wounds, oxidative stress-induced inflammation leads to collagen and ECM degradation as well as impaired angiogenesis [59]. Beyond that, high levels of ROS produced by immune cells in the wound activate nuclear factor-κB and significantly increase expression of the inflammatory mediator interleukin-6 (IL-6) and tumor necrosis factor-α (TNF-α), leading to chronic inflammation and slowing wound healing. Therefore, keeping ROS at the wound site at the appropriate level may help to promote wound healing.

#### Metalloproteinase overexpression

MMPs have a dual role in the progression of diabetic wound healing. MMPs are the gelatin enzyme produced in the dermis after injury and play an important role in ECM disintegration and tissue recombination during healing [60]. However, overexpression of MMPs inhibits the production of early connective granulation tissue to fill the wound and inactivates growth factors critical to the wound-healing process. The overexpression of ECM protease in the wound decreases the accumulation of ECM in DFUs, preventing wound closure and increasing the risk of bacterial infection and chronic inflammation [8, 61]. While scientists have shown that overexpression of MMPs (such as MMP-2 and MMP-9) [62] impedes the wound healing process, other studies have indicated that other MMPs (such as MMP-8) are beneficial for diabetic wound healing [63]. Therefore, selective inhibition of the function of matrix protease is the key to diabetic wound healing. It is worth noting that the level of active MMPs in the wound of diabetic patients might be significantly different from that of diabetic mice used in the experiment. Therefore, these experimental results also suggest that the differences between human and experimental animals should be paid attentions in the design of responsive hydrogel wound dressings.

In short, diabetic chronic wounds have a complicated microenvironment [64] which include excessive exudate [65], hypoxia [66], bacterial infection [67], excess glucose [68], high levels of ROS [69], and overexpression of MMPs [70]. All of these can impede clinical healing in diabetic wounds and represent a challenge that must be overcome in clinical treatment.

## Advantages of smart hydrogel dressings in diabetic wound healing

**Fig. 3:**
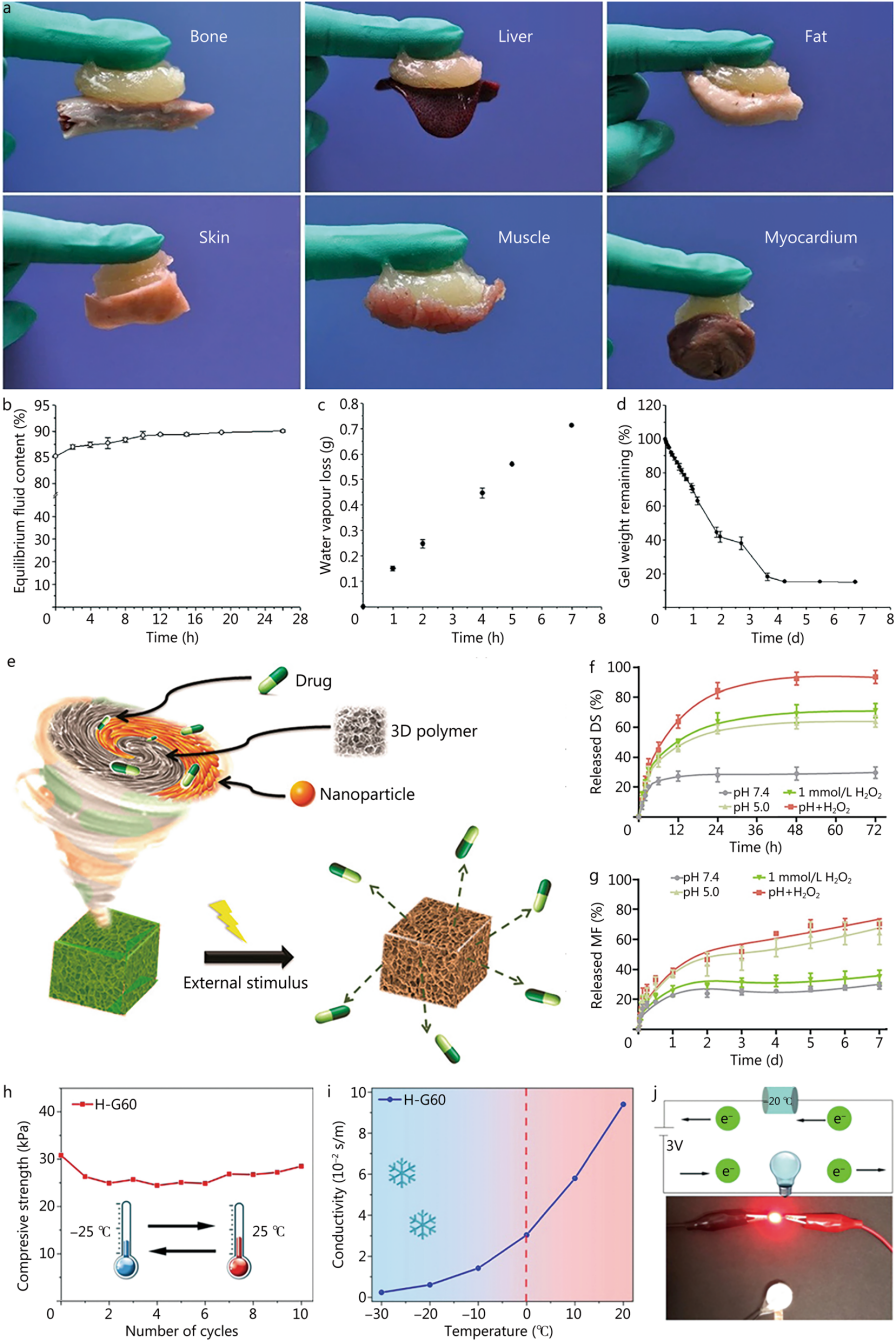
Illustration of the advantages of hydrogels as wound dressings. **a** Images demonstrating adhesion of bioglass (BG)/oxidized sodium alginate (OSA) hydrogel to bone, liver, fat, skin, muscle and cardiac tissue. Reproduced with permission [75]. Copyright 2019, Springer Nature. **b** Equilibrium PBS content of hydrogel. **c** Water vapor loss of hydrogels in a water-rich environment. **d** The remaining weight of the hydrogel during evaporation. Reproduced with permission [77]. Copyright 2005, Elsevier BV. **e** Diagram of a hydrogel with a 3D structure used to load drugs and achieve controlled drug release in response to external stimuli. Reproduced with permission [78]. Copyright 2015, American Chemical Society. Release kinetics of DS (**f**) and MF (**g**) in pH/ROS responsive hydrogel (DS%MIC@MF) under different conditions. Reproduced with permission [83]. Copyright 2022, Elsevier. **h**–**j** Cold resistance of smart hydrogel. **h** Hydrogel H-G60 was successively stored at low temperature and room temperature for 2 h of stress, and was subjected to 10 cyclic stress treatments under the same conditions. **i** Freeze–thaw cycle curve of H-G60 at − 20 °C. **j** The hydrogel is connected to the circuit at − 20 °C for 2 h and the light emitting diode (LED) bulb is very bright. Reproduced with permission [84]. Copyright 2022, American Chemical Society. 3D three dimensions, DS diclofenac sodium, MF mangiferin

Wound dressing is a major part of diabetic wound treatment, and the ideal wound dressing should be able to effectively absorb wound exudate while providing a microenvironment that facilitates wound healing [35]. Traditional wound dressings, such as gauze, bandage and other inert dressings, are the most used wound dressings with their simple production and low cost. They can absorb exudate, have a certain protective effect on the wound, and can be safely combined with antibiotics. However, traditional wound dressings do not promote wound healing. In addition, traditional dressings tend to form a dry scab with the wound that can cause secondary damage to the wound during the process of removal, causing pain and other discomforts to the patient [71, 72]. Recently, wound dressings such as films, foams, hydrocolloids, alginates, and hydrogels [19, 73, 74] have been developed based on the theory of healing in wet environments, and researchers have found that the wet environment is more conducive to wound repair. Film dressings have excellent breathability while also isolating fluids and bacteria that are suitable for superficial wounds with less exudation. Foam dressings, including Allevyn (Smith and Nephew) and Cavicare (Smith and Nephew), keep the wound warm and hydrated and prevent secondary damage to the wound when removed. Hydrophilic adhesive dressings [such as Duoderm (Convatec), Granuflex (Convatec), and Comfeel (Coloplast)] and sodium alginate (SA) dressings [Kaltostat (Convatec) and Sorbsan (Maersk Medical)] are popular wound dressings that have excellent exudate absorption properties, but their use with infected wounds is controversial. There are also a number of commercially available hydrogel dressings, including Aquaform (Maersk Medical) and Intrasite Gel (Smith and Nephew). The bioadhesive properties [75, 76] (Fig. 3a), excellent water absorption properties [21, 77] (Fig. 3b–d), three-dimensional porous structure [78] (Fig. 3e), and adjustable degradation rate [79] of hydrogel dressings make them suitable for the delivery of drugs and bioactive substances.

Hydrogel dressings have been extensively studied in diabetic wound healing in the past few decades [80]. Hydrogel dressings are usually made of natural or synthetic polymers and have advantages such as biodegradability over traditional wound dressings such as bandages. Therefore, for the increasing number of diabetic foot patients, the promotion and application of hydrogel dressings might be able to alleviate environmental pressure to a certain extent. In addition, self-adhesive wound dressings on the market are mainly composed of non-woven fabric backing, polypropylene bonding agent, silicone wound bonding layer and polyethylene film cover. While hydrogel dressings with bioadhesion do not need to add adhesives, and their components are simpler and more biocompatible. Especially, the development and in-depth research of smart hydrogel dressings with special properties (for example, stronger bioadhesive ability [81], controlled drug delivery and adjustable environmental adaptability) that provide a sustainable platform and opportunity for diabetic wound healing [82]. In this section, we introduce the advantages of smart hydrogels for the treatment of diabetic wounds.

### Controllable drug delivery

The effective delivery of drugs or other small molecules in dressings is a crucial concern for wound healing effectiveness. Based on the strong bioadhesive properties of biomaterials, the response of hydrogels to stimuli such as MMPs, ROS, pH and exogenous light [85] can achieve efficient delivery and controlled release of active ingredients. The therapeutic effect of exosomes (EXO) on wound healing has been researched for many years, but the diabetic wound microenvironment limits the delivery and release of EXO with conventional hydrogel dressings, limiting their therapeutic effect [86]. More recently, smart hydrogel dressings that use MMP enzyme response have been developed that improve the delivery of EXO, especially the efficient delivery and controlled release of adipose-derived stem cell exosomes (ADSC-exo; providing sustained release for 20 d with 90% release), thereby boosting the therapeutic effect of ADSC-exo [87].

Diabetic wounds are low pH environments. Schiff-base cross-linking is the classical reaction mode for polymeric smart hydrogel formation which can achieve controlled drug release under pH stimulation [88]. A pH-responsive smart hydrogel dressing based on ethylenediamine-modified gelatin (N-Gel) with oxidized dextran (ODex) containing rich aldehyde groups was constructed at pH 8.5 by Guo et al*.* [89] to respond to the low pH and overexpression of ROS present in chronically infected wounds for effective drug delivery and controlled release. Inspired by dual-response hydrogels, Wu et al*.* [83] developed hydrogels based on a dual pH/ROS response that can achieve the spatiotemporal release of different drugs (Fig. 3f, g).

The development of exogenous stimulus-responsive smart hydrogel dressings to achieve controlled drug release has been a hot research topic in recent years [90]. Using the light-responsive properties of graphene oxide (GO), researchers have constructed a smart hydrogel dressing with high mechanical strength formed by GO-containing benzaldehyde and cyanoacetate dextran solutions with histidine. A light-responsive smart hydrogel was developed by Ye et al*.* [91] that includes rifampicin hydrogel dressing with a hollow structure and good biocompatibility with natural halloysite clay nanotubes (HNTs), encapsulated with fatty acids and indocyanine green (ICG). With near-infrared (NIR) laser irradiation, the ICG locally heats the fatty acid by thermal conversion after the melting point is surpassed, and the nanotubes slowly release rifampicin, causing an antimicrobial effect in the wound. The excellent photothermal properties and strong mechanical properties conferred by this type of hydrogel make it possible to manage the sustained release of the drug. Diabetic wound healing is a complex process, and dressings and medications are common combinations, making this type of controlled and effective drug delivery particularly important.

### Adjustable environmental adaptability

Currently available medical hydrogel dressings have a single mechanical property and are unable to adjust to the changing environmental stimuli, which often decelerates the healing process [41]. A smart, multifunctional hydrogel dressing possesses robust moisturization, long-lasting antibacterial properties and adaptability to changing environmental conditions. Dong et al*.* [65] designed a wound dressing based on SA and poly[2-(methacryloyloxy)-N,N,N-trimethylethylamine ammonium chloride] that not only has a water retention rate of more than 90% for 7 d but also has excellent anti-freezing properties. It has good electrical conductivity at temperatures down to − 20 °C and mechanical properties that show outstanding environmental adaptability and stability. Therefore, it might be a more suitable delivery vehicle for drugs that require cryopreservation. Moreover, the SA and [2-(methacryloyloxy)ethyl]dimethyl-(3-sulfopropyl)-based smart hydrogel dressing developed by the team has excellent antimicrobial adhesion and stability while adapting to cold environments (Fig. 3h–j) [84]. Chen et al*.* [92] prepared a hydrogel that is stretchable (up to 2167%), conductive (17.1 mS/cm), strongly adhesive (up to 0.28 MPa on glass), and self-healing (90% efficient recovery). The hydrogel is suitable for use as a biosensor to monitor human activity, and might also be adapted to monitor the dynamics of wounds. In addition, ROS- and glucose-responsive [93, 94] hydrogel wound dressings not only respond to the environment but also consume ROS and glucose, thereby improving the microenvironment at the wound site.

## Potential application of responsive hydrogel dressing for diabetic wound healing

Currently, many gel products for the treatment of diabetic wounds have completed, or are undergoing clinical trials (Table 1). Thus, the potential of the hydrogel dressings for the treatment of diabetic wounds can be further explored. Clinically, hydrogel dressings are relatively consistent in terms of degradation and drug release. However, individual wound states and environments differ, which may lead to differences in therapeutic effects. Therefore, many studies have taken advantage of hydrophobic interactions or introduced reversible covalent chemical bonds (such as Schiff-base bonds, disulfide bonds, borates ester bonds, acylhydrazone bonds, and Diels–Alder reactions) into hydrogels to obtain injectable hydrogel dressings that can respond to glucose, pH, ROS, temperature, and enzyme stimulation. These responsive-hydrogel wound dressings can regulate self-degradation and other behaviors, allowing the precise control of drug release in specific environments, which provides a reference for the design of individualized clinical treatment programs. Of course, the premise of personalized treatment is to accurately assess the individual wound environment. This section introduces a variety of smart responsive hydrogel wound dressings with potential for clinical diabetes wound treatment (Table 2) [95–109].

**Table 1:** Hydrogel dressings for diabetic wounds in completed or ongoing clinical trials from the National Library of Medicine

Hydrogel dressing	Conditions or diseases	Clinical phases	Status	Clinical trials numbers	Date	Marketed
AmeriGel^®^	Type 1 diabetes; Type 2 diabetes; DFUs	4	Terminated (study closed due to recruitment problems)	NCT01350102	2012.04–2014.03	No
Fitostimoline^®^ hydrogel	Diabetic foot	4	Completed	NCT05661474	2021.02–2022.12	Yes
SANTYL^®^	DFUs; Diabetic foot wounds	4	Completed	NCT02111291	2014.04–2015.12	Yes
Woulgan Gel	DFU	4	Completed	NCT02631512	2015.10–2019.04	Yes
ALLO-ASC-DFU (hydrogel sheet containing allogenic mesenchymal stem cells)	DFU	1	Completed	NCT03183726	2016.01–2017.07	No
Hydrogel Purilon^®^	Diabetic foot; Diabetic Neuropathy; Foot ulcer	2	Completed	NCT03700580	2012.08–2016.10	No
IZN-6D4 Gel	DFU	2	Completed	NCT01427569	2012.03–2015.08	No
Lavior Diabetic Wound Gel	DFU	2	In progress	NCT05607979	2022.12–2023.06	No
NanoDOX™ Hydrogel	DFU	2	Completed	NCT00764361	2009.01–2010.08	No
TWB-103 (mixture of TWB-102 cell and TWB-103 hydrogel)	DFU	1/2	Unknown	NCT03624023	2019.12–2021.07	No
Hydrogel with 3% sodium pentaborate pentahydrate	Wound healing	1	In progress	NCT02241811	2014.09–2023.12	No
RMD-G1 (a hydrogel containing erythropoietin)	DFU	1	Completed	NCT02361931	2016.03–2018.06	No
Cadexomer iodine gel	DFU	Not applicable	Terminated (recruitment challenges)	NCT02181621	2014.08–2015.10	No
Hydrogel/nano silver-based dressing	Diabetes mellitus	Not applicable	Completed	NCT04834245	2019.01–2019.12	No
Regranex^®^	DFU	Not applicable	Unknown	NCT00446472	2007.04–2010.09	No
Solosite gel	DFU	Not applicable	Terminated	NCT02181621	2014.08–2015.10	No
ConvaTec DuoDERM Hydroactive Gel	DFU	Not applicable	Completed	NCT00971048	2009.09–2011.01	No

*ASC* allogenic mesenchymal stem cells, *DFU* diabetic foot ulcer, *NCT* National Clinical Trial

**Table 2:** Mechanism and composition of responsive hydrogel wound dressing for diabetes

Responsivity	Mechanism	Therapeutic agent	Materials	References
pH	Acylhydrazone bond and imine bond	Insulin	N-carboxyethyl chitosan, dihydrazine adipate and hyaluronic acid–aldehyde	[95]
	Imine bond	Ceftazidime	ODex and AMP DP7 (VQWRIRVAVIRK)	[27]
		AgNPs and pro-angiogenic drug DFO	Dopamine, ODex polymers	[96]
		Umbilical cord stem cell factor	Collagen- and aldehyde-modified PEG	[97]
	Exchange of sodium and calcium ions	Protamine nanoparticles and hyaluronan oligosaccharides	Na-alginate and Ca^2+^ ions	[98]
	Hydrogen bond and hydrazone bond	Metformin	Hyaluronic acid and collagen	[99]
ROS	Borate ester bond	Mupirocin, granulocyte-macrophage colony-stimulating factor	N^1^, N^1^, N^3^, N^3^-tetramethylpropane-1, 3-diaminium and PVA	[25]
	Hydrogen bond	No	Quaternized chitosan and TA	[100]
	Phenylboronic ester bond	No	PPBA, TA and PVA	[93]
	Phenylboronic ester bond	Doxycycline hydrochloride	Sodium alginate and sodium hyaluronate modified by 3-aminophenylboric acid and PVA	[101]
Enzyme	MMP degradable peptide	ADSC-exo	Four-arm PEG functionalized by maleimide, the substrate peptides of MMP [MMP(W)x] and PFG-dithiol	[87]
	Metal chelating and hydrogen bond	Curcumin	L-carnosine (b-alanyl-L-histidine) and silk fibroin	[102]
Glucose	Borate bonds	Insulin	Gelatin methacrylate, 4-(2-acrylamide ethyl aminoformyl)-3-fluorobenzene boric acid and gluconic acid (G-insulin)	[103]
	GOX	Deferoxamine mesylate	IDA, DFO, Zn(NO_3_)_2_·6H_2_O, and GOX	[94]
	Phenylboronic ester bonds	No	PEG-DA and PBA modified hyaluronic acid	[104]
	Phenylboronic ester bonds	No	PBA modified hyaluronic acid methacrylate and catechin	[105]
pH/ROS	Amide bond and imine bond, mercaptan group	Zinc oxide nanoparticles, paeoniflorin	Ethylenediamine-modified gelatin and oxidized dextra	[89]
	Boronic ester bonds and imine bond	Diclofenac sodium, mangiferin	Phenylboronic acid grafted oxyglucan and caffeic acid grafted ε-polylysine	[83]
	Phenylboronic ester bonds	Vancomycin-conjugated silver nanoclusters, nimesulide	Phenylboronic acid-modified gel and PVA	[106]
pH/glucose	Benzoic-imine bonds and phenylboronic ester bonds	Insulin and fibroblasts	Phenylboronic-modified chitosan, PVA and benzaldehyde-capped poly(ethylene glycol)	[107]
	Imine bond and phenylborate bond	Metformin and graphene oxide	PEG-modified with formyl benzoic acid and PBA, Dihydrocaffeic acid and L-arginine grafted chitosan	[26]
pH/temperature	AAc, hydrophobic interaction	Ultrasmall AgNPs	N-PNIPAM and AAc	[22]
	Imine bond, hydrophobic interaction	The adipose mesenchymal stem cells -derived exosomes	Pluronic F127, oxide hyaluronic acid and poly-ε-L-lysine	[66]
	Sulfonamide groups, hydrophobic interaction	No	Poly(sulfamethazine ester urethane), PEG	[108]
Temperature/enzyme	Gelatin (substrate of MMP-9), hydrophobic interaction	Curcumin	Gelatin and Pluronic F127	[70]
Photo/magnetic	MNPs@MXene, hydrophobic interaction	*AgNPs*	Fe_3_O_4_@SiO_2_ magnetic nanoparticles, PNIPAM and alginate	[109]

*AAc* acrylic acid, *ADSC-exo* adipose-derived stem cell exosomes, *AgNPs* silver nanoparticles, *AMP DP7* antimicrobial peptide DP7, *DFO* deferoxamine, *GOX* glucose oxidase, *IDA* 4,5-imidazoledicarboxylic acid, *MMP* matrix metalloproteinase, *MNPs@MXene* Fe_3_O_4_@SiO_2_ magnetic nanoparticles, *N-PNIPAM* N-isopropylacrylamide, *ODex* oxidized dextran, *PEG* polyethylene glycol, *PEG-DA* poly (ethylene glycol) diacrylate, *PVA* poly(vinyl alcohol), *TA* tannic acid, *PPBA* phenylboronic acid-modified polyphosphazene, *ROS* reactive oxygen species

### Glucose-responsive hydrogel dressing

**Fig. 4:**
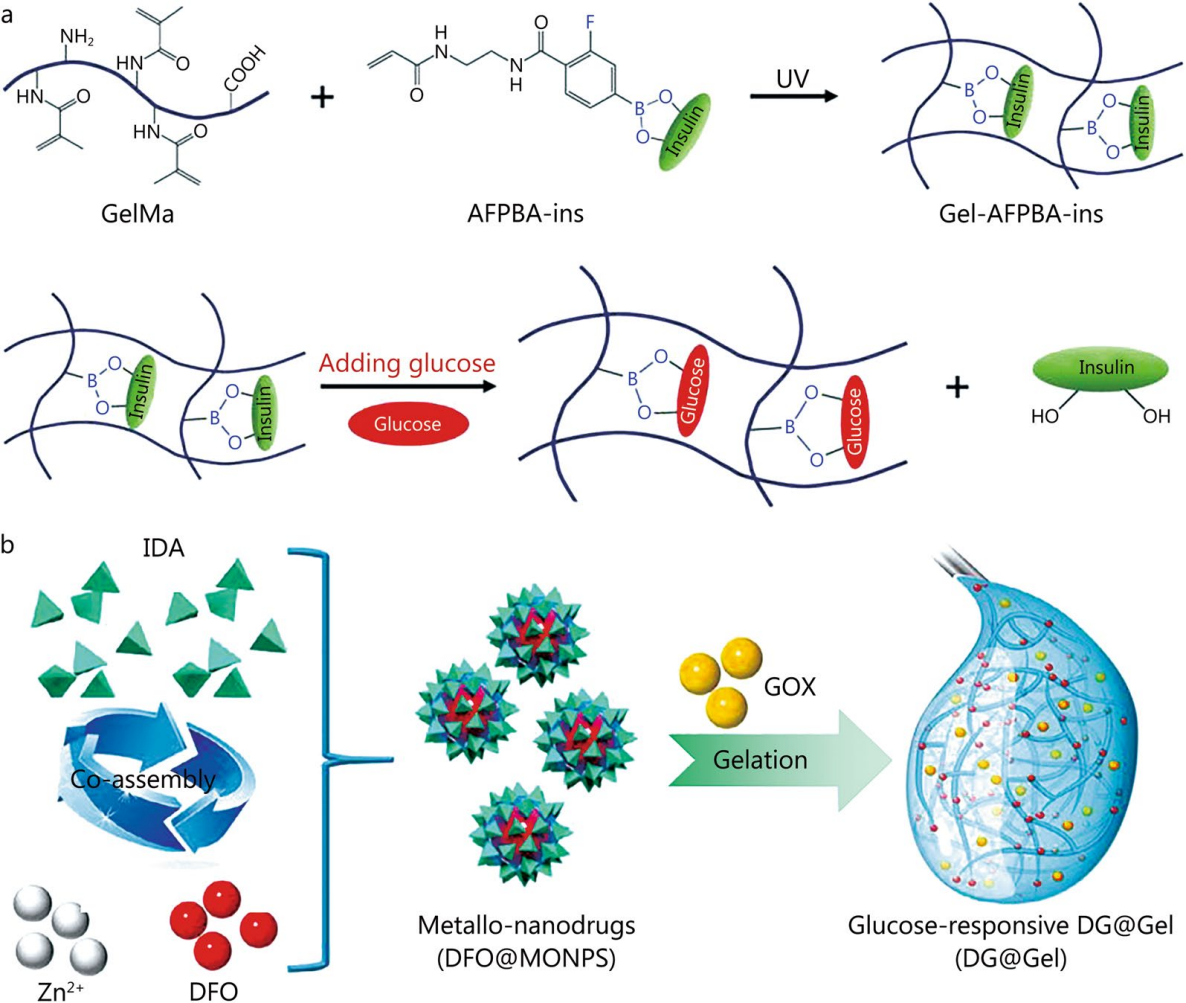
Construction process and the structure of representative glucose-responsive hydrogels. **a** Diagram of the preparation process of Gel-AFPBA-ins hydrogels to achieve glucose-responsive release of insulin and the process of releasing insulin in a glucose environment. Reproduced with permission [103]. Copyright 2022, Royal Society of Chemistry. **b** Diagram of the synthesis of a glucose-responsive injectable hydrogel based on GOX with zinc ions, organic ligands, and a small-molecule drug, DFO mesylate. Reproduced with permission [94]. Copyright 2022, American Chemical Society. AFPBA-ins 4-(2-acrylamide ethyl aminoformyl)-3-fluorobenzene boric acid with insulin, DG@Gel glucose-responsive multifunctional metal–organic drug-loaded hydrogel, DFO deferoxamine, GOX glucose oxidase, GelMa gelatin methacrylate, Gel-AFPBA-ins the hydrogel system was composed of GelMa and AFPBA-ins, IDA 4,5-imidazoledicarboxylic acid, UV ultraviolet

Hyperglycemia is the main cause of the diabetic wound complex microenvironment and a major obstacle to clinical diabetic wound treatment. By considering the hyperglycemic microenvironment inherent in diabetic wounds, a number of glucose-responsive hydrogel wound dressings were designed to improve wound microenvironment and achieve controlled drug release, which might become in major demand for clinical diabetic wound dressing. In details, glucose-responsive hydrogels can undergo phase transformation in response to changes in glucose concentration in the environment. With the progress of insulin-controlled release study, glucose-responsive hydrogels have become a popular topic that can be classified into three areas: (1) hydrogels loaded with glucose oxidase (GOX); (2) a hydrogel system containing concanavalin A (Con A); and (3) a system containing a phenylboronic acid group (PBA). GOX and Con A are naturally sourced proteins with excellent glucose selectivity. While PBA is a fully synthetic receptor and can bind to monosaccharides containing cis-diol groups. These glucose-responsive hydrogels have been developed as smart platforms for the controlled release of therapeutic agents such as insulin in response to glucose levels [110]. Insulin, a peptide hormone commonly used by diabetics to lower blood sugar, has widely been utilized to promote wound healing in glucose-responsive hydrogel dressing. It is worth mentioning that the controlled release of insulin may have a better hypoglycemic effect than the explosive release of insulin in diabetic patients. As a typical example, Guo et al*.* [103] prepared hydrogels with unique glucose-responsive phenylborate groups by in situ copolymerization of gelatin methacrylate, glucose-responsive monomer 4-(2-acrylamide ethyl aminoformyl)-3-fluorobenzene boric acid (AFPBA) and gluconic insulin (G-insulin) (Fig. 4a). This hydrogel-based microneedle dressing exhibited a glucose-responsive insulin release behavior that reduced inflammation, enhanced collagen deposition, and improved the hyperglycemic environment in diabetic wounds, expediting the wound healing process of type I diabetic mice (C57BL/6) induced by streptozocin (STZ).

Although PBA-mediated glucose-responsive hydrogels are stable in composition, PBA is less selective to glucose than the natural proteins GOX and Con A [110]. GOX can catalyze the production of glucuronic acid and hydrogen peroxide from glucose, resulting in decreased pH and antibacterial activity. For example, Yang et al*.* [94] reported on an injectable, “self-healing”, glucose-responsive, drug-loaded metal–organic hydrogel (DG@Gel) with zinc ions that overcame bacterial resistance to antibiotics. More interestingly, this hydrogel had synergistic antibacterial action with hydrogen peroxide catalyzed by GOX, while the loaded drug [deferoxamine (DFO)] could promote the formation of blood vessels and reduce excess free radicals in wounds (Fig. 4b). The pH of the solution in the DG@Gel group fell dramatically as glucose content increased and the release of DFO was expedited. Therefore, DG@Gel could improve the hyperglycemic microenvironment of the wound, with antibacterial, anti-inflammatory, and pro-angiogenic abilities, thereby facilitating diabetic wound healing in type I diabetic mice (BALB/c). It should be noted that GOX-based glucose-responsive hydrogel dressings produce gluconic acid by consuming glucose, which might result in a significant pH change at the wound site, while pH also plays an important role in the wound healing process. Therefore, clinical use of GOX-based hydrogel dressing might require regular monitoring of wound pH or mitigation measures. Con A-based glucose-responsive hydrogels are often developed to control insulin release [111–113]. However, most of the relevant studies are in vitro experiments, and there are few studies on diabetic wound models. The binding mode of Con A to glucose is competitive binding, relatively unstable, coupled with its poor biocompatibility, which limits its further development. As hyperglycemic environment is always an obstacle in the treatment of diabetic wounds, the physiological functions of the existing clinical hydrogel dressings are weak and unresponsive to the complex microenvironment, leading to poor efficacy. Glucose-responsive hydrogel dressings not only have basic drug-loading functions but also improve the microenvironment to achieve synergistic therapeutic effects. However, specific modifications are needed to produce different glucose responsive hydrogels. For example, PBA-based hydrogels need to improve the selectivity of PBA to glucose, while protein-based hydrogels (GOX and Con A) need to ensure protein stability.

### pH-responsive hydrogel dressing

Hydrogel dressings that are pH-responsive can provide on-demand release of drugs, such as antibiotics and insulin, which are able to maintain drug concentrations in specific situations, reduce side effects, and improve treatment efficiency [114]. The pH fluctuation throughout different stages of wound healing may be related to the progress of wound healing [14, 115], making pH a natural indicator for drug release.

**Fig. 5:**
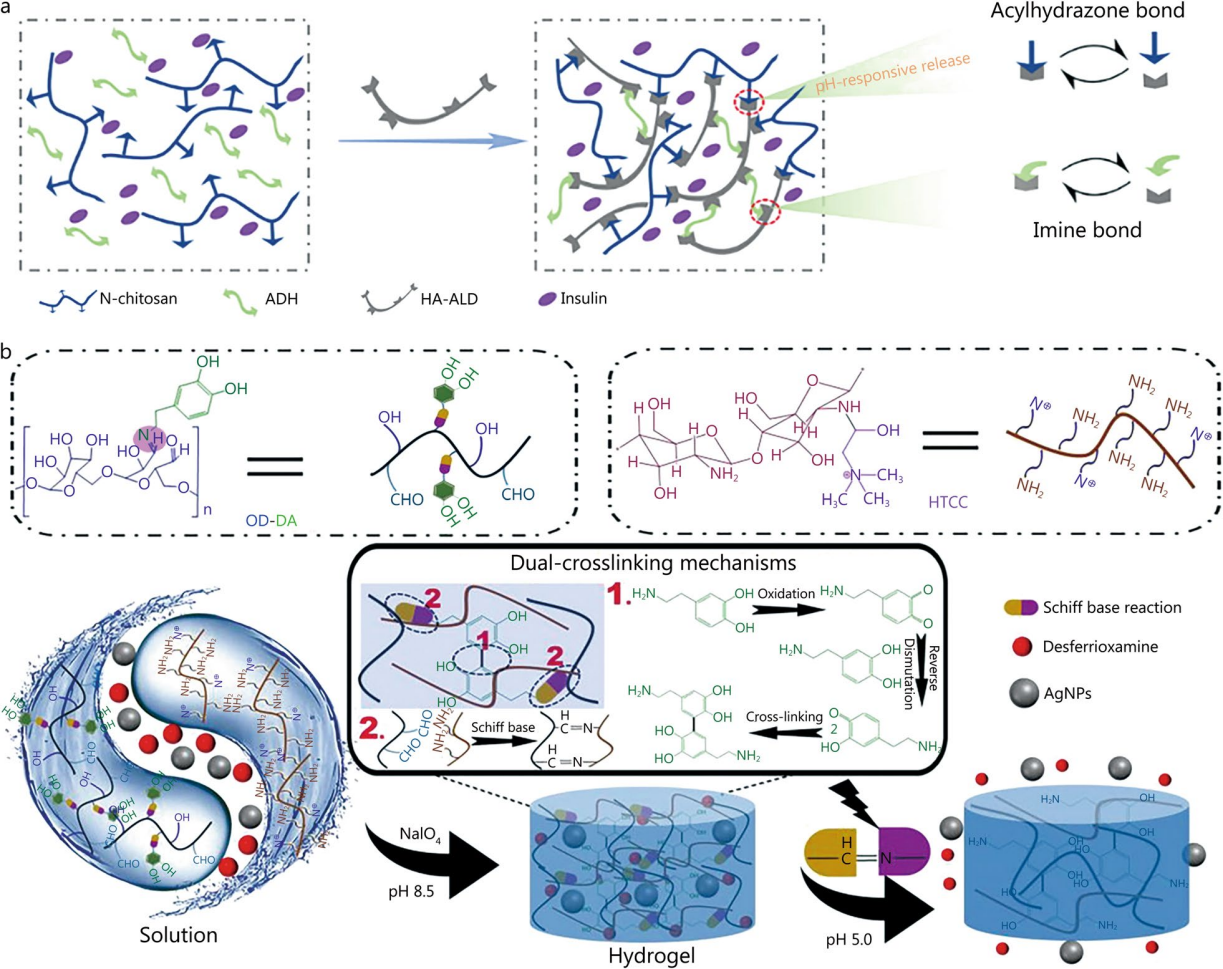
Construction process and structure of representative pH-responsive hydrogels. **a** Synthesis of insulin-loaded pH-responsive hydrogels based on acylhydrazone bonds. Reproduced with permission [95]. Copyright 2021, Elsevier. **b** Mechanism of pH-responsive hydrogel synthesis based on double Schiff-base bond and schematic diagram of the process of releasing AgNPs and deferoxamine under acidic conditions. Reproduced with permission [96]. Copyright 2021, Elsevier. ADH adipic acid dihydrazide, AgNPs silver nanoparticles, HA-ALD hyaluronic acid-aldehyde, N-chitosan N-carboxylethyl chitosan, OD-DA oxidized dextran-dopamine, HTCC chitosan quaternary ammonium salt

Hyaluronic acid (HA) is a naturally derived polysaccharide commonly used as a wound dressing because of its potential to promote cell migration and associated signaling. Lee et al*.* [18] used hyaluronic acid dressing (Healoderm) to treat DFUs and showed that pure HA could promote wound healing without other side effects. With the development of multifunctional drug-loaded hydrogel dressings, Li et al*.* [95] designed a hydrogel composed of N-carboxylethyl chitosan (N-chitosan) and adipic acid dihydrazide (ADH), with in situ cross-linked HA-aldehyde (HA-ALD) (Fig. 5a). Insulin glargine was incorporated into the hydrogel to achieve sustained release and pH responsiveness for up to 14 d. Hydrogel disintegration increased when the pH was decreased from 7.4 to 6.5, resulting in an accelerated release of insulin from the hydrogel. The results of wound treatment in diabetic rats showed that the hydrogel dressing shortened the inflammatory period, accelerated wound healing and improved peripheral neuropathy.

Diabetic wound infection is a common problem, and silver nanoparticles (AgNPs) are often used in clinical antibacterial hydrogel dressings in addition to antibiotics. For example, the Aquacel Ag dressing for clinical use combines 1.2% silver with Aquacel hydrofiber and distributes it throughout the dressing, enabling the slow release of silver at the wound site for up to 2 weeks, enhancing the bactericide effect [116]. However, its drug-release behavior cannot be adjusted according to different wound environments. To achieve a controlled release of medication at the wound site, Hu et al*.* [96] designed a double-cross-linked pH-responsive hydrogel encasing AgNPs and the angiogenic drug DFO (hydrogel@AgNPs&DFO) with demonstrated antibacterial and angiogenic properties (Fig. 5b). In acidic, bacterially infected diabetic wounds, double Schiff-base cracking, chitosan quaternary ammonium salt and the release of AgNPs can swiftly kill bacteria on the wound site and reduce inflammation, while DFO promotes angiogenesis at the wound site. The release rate of the hydrogel at pH 5.0 was faster than that at pH 7.4, and in streptozocin-induced type 2 diabetic rats, wounds infected with *Staphylococcus aureus* (*S. aureus*) were completely healed after 10 d, while wounds in the control group took 17 d to heal.

For multifunctional wound dressings, drug release has a great impact on wound treatment effect. With the help of pH changes in the wound environment, drug release on demand can be accurately achieved, avoiding frequent drug administration or the formation of drug resistance in clinical practice. However, this also presents new challenges to the production of wound dressings. On the one hand, the pH of the dressing itself should be appropriate to avoid causing new irritation to the wound. On the other hand, it should have appropriate sensitivity to pH responses at the wound site to ensure the normal release of drugs.

### ROS-responsive hydrogel dressing

**Fig. 6:**
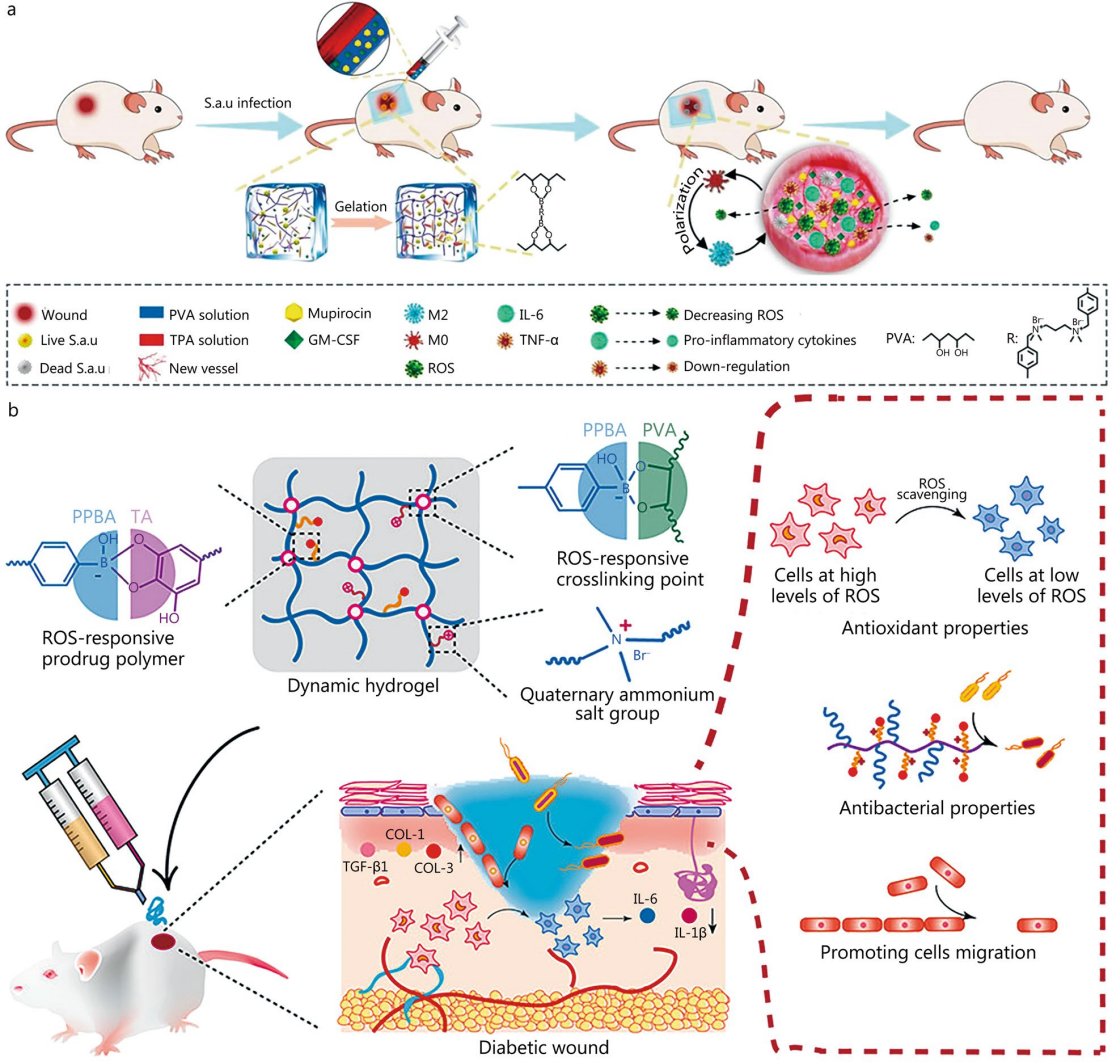
Construction process and the structure of representative ROS-responsive hydrogels. **a** The formation and mechanism of a PVA-based hydrogel cross-linked with an ROS-responsive linker loaded with therapeutics for the treatment of wounds with bacterial infection. Reproduced with permission [25]. Copyright 2020, Elsevier. **b** Schematic synthesis of ROS-responsive hydrogel PPBA-TA-PVA and its anti-inflammatory mechanism in diabetic wound sites. Reproduced with permission [93]. Copyright 2022, American Chemical Society. COL-1 collagen-1, COL-3 collagen-3, GM-CSF granulocyte-macrophage colony-stimulating factor, IL-1β interleukin-1β, IL-6 interleukin-6, PPBA phenylboric acid-modified polyphosphazene, PVA polyvinyl alcohol, ROS reactive oxygen species, S.a.u *Staphylococcus aureus*, TNF-α tumor necrosis factor-α, TA tannic acid, TPA N1-(4-boronobenzyl)-N3-(4-boronophenyl)-N1, N1, N3, N3-tetramethylpropane-1, 3-diaminium, TGF-β1 transforming growth factor beta 1

High ROS levels are a key factor hindering the healing of diabetic wounds, and they are also a hot topic in the research of responsive hydrogels. Zhao et al*.* [25] developed an ROS-scavenging hydrogel that responds to high concentrations of ROS in the wound microenvironment and is loaded with antibiotics and granulocyte-macrophage colony-stimulating factor (GM-CSF), a growth factor that promotes tissue regeneration (Fig. 6a). The hydrogel dressing can significantly reduce intracellular ROS levels, reduce pro-inflammatory factor secretion, regulate macrophage phenotype change, promote the formation of new blood vessels and collagen, and significantly improve wound healing ability. Quaternized chitosan (QCS) is a positively charged polysaccharide derivative of chitosan that exhibits inherent antibacterial action and is often utilized as an antibacterial material [117]. Tannin is a bioactive substance that promotes clotting as well as a tiny natural molecule with a high phenolic hydroxyl content that may cleanse ROS. Pan et al*.* [100] prepared an injectable hydrogel (QT) from QCS and tannic acid (TA) that showed high hemostatic, antibacterial and ROS clearance properties, and minimized blood loss in rats undergoing surgical tail resection. Due to the oxidation of TA, QT with H_2_O_2_ becomes a solution state after 12 h, which contributes to the release of active components in the hydrogel network. Moreover, in diabetic rat skin models, the QT treatment group showed faster collagen deposition and accelerated skin tissue regeneration. Ni et al*.* [93] also made use of the antioxidant properties of TA, combined with phenylboronic acid-modified polyphosphazonitrilene (PPBA) and polyvinyl alcohol (PVA), and obtained a hydrogel (PPBA-TA-PVA) with an ROS-responsive and anti-inflammatory function (Fig. 6b). The dynamic phenylborate connection gives PPBA-TA-PVA hydrogels injectable and self-healing capabilities that can be tailored to irregular deep wounds, allowing the hydrogels to successfully react to mechanical traumas in constantly moving joint wounds. In vivo experiments showed that, in comparison with commercially available Tegaderm films, PPBA-TA-PVA hydrogel could significantly shorten the inflammatory period of STZ-induced rat diabetic wounds and accelerate wound healing. The regulation of wound microenvironments is crucial for the treatment of difficult-to-heal diabetic wounds, where excess ROS is believed to be a key factor in the delayed repair of diabetic wounds and can induce infection. ROS-responsive hydrogel dressings can not only achieve controlled release of drugs but also reduce ROS levels at the wound site to improve the wound microenvironment. However, it is also worth considering how to maintain normal ROS levels in diabetic wounds, because certain levels of ROS can kill bacteria and promote wound healing.

### Enzyme-responsive hydrogel dressing

**Fig. 7:**
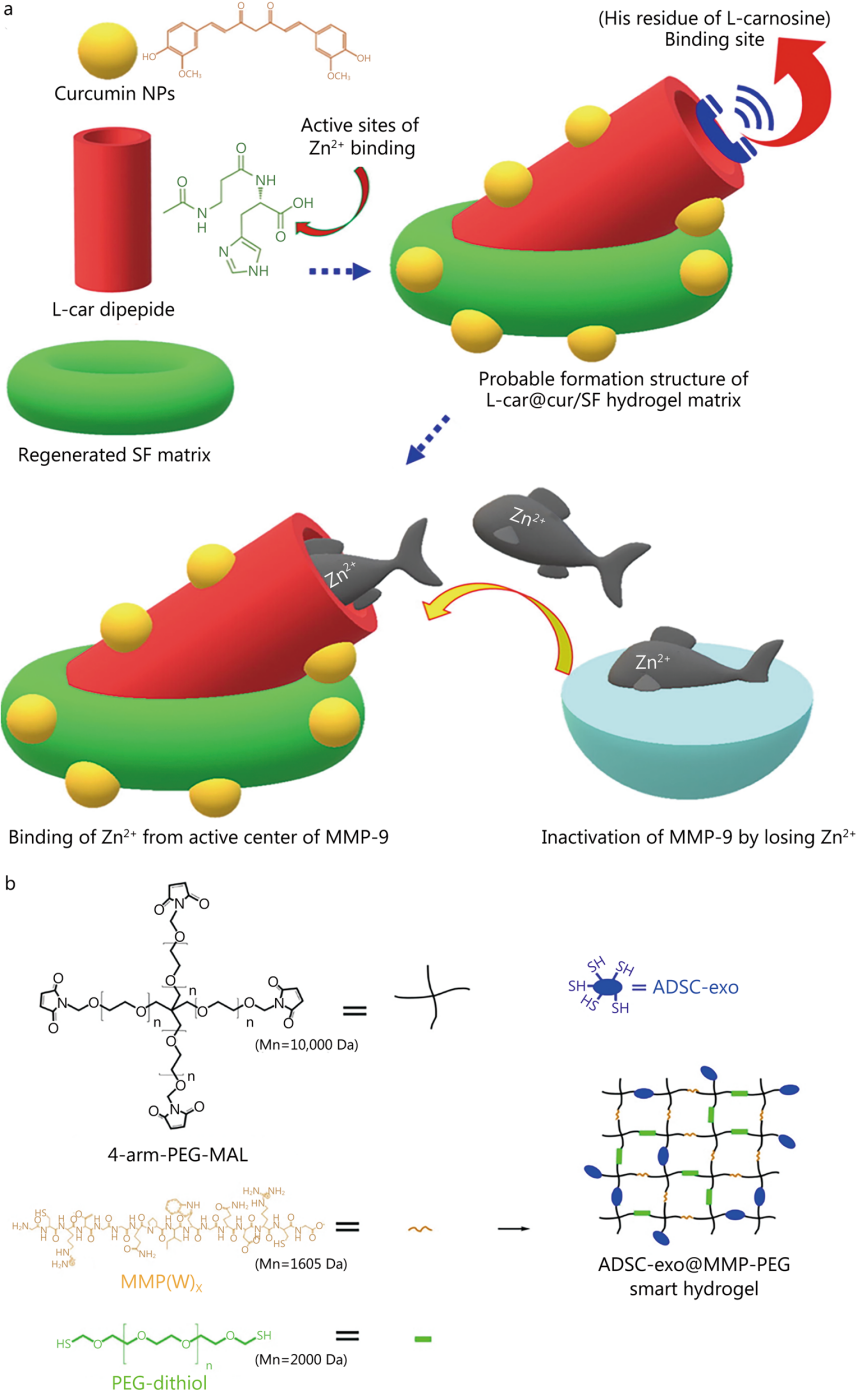
Construction process and the structure of representative enzyme-responsive hydrogels. **a** Composition structure of biological hydrogels based on L-carnosine and the principle of inhibiting matrix metalloproteinases. Reproduced with permission [102]. Copyright 2020, Elsevier. **b** Composition and synthesis of ADSC-exo@MMP-PEG smart responsive hydrogels. Reproduced with permission [87]. Copyright 2022, Elsevier. ADSC-exo adipose-derived stem cell exosomes, MAL maleimide group, MMP matrix metalloproteinase, NPs nanoparticles, PEG polyethylene glycol, SF silk fibroin, L-car L-carnosine

Compared with ordinary wounds, diabetic wounds overexpress MMPs, which delays the process of wound healing. An MMP-responsive hydrogel dressing can induce MMPs inactivation or protect cells through substrate competition, a potentially feasible method for clinical acceleration of wound healing. Knowing that Zn^2+^ can positively affect the activity of MMP-9 [118], Sonamuthu et al*.* [102] developed curcumin and L-carnosine loaded silk fibroin (SF) composite hydrogel (L-car@cur/SF). L-carnosine suppresses MMP-9 activity by chelating Zn^2+^ in the active center of MMP and together with curcumin relieves the inflammation of the diabetic wound site, effectively promoting wound healing in diabetic mice (Fig. 7a). EXO, which are released by some cells (such as mesenchymal stem cells, fibroblasts, and immune cells), can enhance wound healing. For example, Wang et al*.* [119] discovered that ADSC-hEVs regulate fibroblast proliferation and ECM formation by activating the PI3K/Akt pathway, which accelerates the healing of diabetic wounds. Jiang et al*.* [87] designed a smart hydrogel (ADSC-exo@MMP-PEG) that responds to MMP-2 formed at room temperature from four-arm polyethylene glycol (PEG) functionalized by maleimide, the substrate peptides of MMP [MMP(W)x], a PEG chain functionalized by a sulfhydryl group (PEG-SH), and ADSC-exo formed by a Michael addition reaction (Fig. 7b). For up to 20 d in the presence of MMP-2, the hydrogel progressively degrades, providing a slow controlled release of ADSC-exo, improving cell proliferation and migration via the Akt pathway. In vivo studies have shown that ADSC-exo@MMP-PEG promotes wound repair in diabetic mice by promoting re-epithelialization, collagen deposition, cell proliferation, and neovascularization. Enzyme-responsive hydrogel dressings can ameliorate overexpression of MMPs in diabetic wounds and be removed at any time according to the degree of wound healing, making them safe. In addition, enzyme catalysis is specific and efficient, enabling more precise and controlled drug release. However, factors such as wound microenvironment and dressing pH may affect the catalytic activity of enzymes, which is a problem requiring special attention in dressing design, production and clinical use.

### Temperature-responsive hydrogel dressings

Temperature is closely related to wound healing because many enzyme response rates are temperature-dependent, while temperature is also a classic indicator of the clinical signs and symptoms checklist used to evaluate chronic wounds and might also serve as a trigger for responsive wound dressings [120, 121]. Lin et al*.* [122] examined, recorded, and analyzed the temperature of the wound bed and surrounding skin in 50 patients with pressure ulcers, and found that a higher periwound temperature facilitated wound healing. Fierheller et al*.* [121] monitored the skin temperature around wounds in 40 patients with chronic leg ulcers and found that the average temperature increased by more than 2 F when wounds were infected. Temperature-responsive hydrogels can shift between solution and glue phases as temperature changes and have improved adaptability to uneven wounds. Furthermore, loaded pharmaceuticals can be released throughout the shrinking process of temperature-sensitive hydrogels to enable regulation of drug release. Polymer solutions with an upper critical solution temperature (UCST) shrink by cooling below UCST in a positive thermo-reversible system, whereas negative thermo-reversible hydrogels with a lower critical solution temperature (LCST) shrink by heating above the LCST [123]. For example, Zhang et al*.* [124] designed a temperature-responsive hydrogel dressing, GelMA-PDA-ASP, which can achieve controlled release of drugs and is significantly superior to traditional dressings, commercial 3 M or gelatin dressings, in promoting wound healing in mice. Heilmann et al*.* [125] selected Poloxham 407 25 wt% hydrogel as a morphine carrier for the treatment of severe skin wounds. This hydrogel dressing releases medication for up to a day, allowing continuous pain relief and avoiding frequent dressing changes. More importantly, temperature-responsive hydrogel dressing can not only respond to the temperature of human skin, but also be regulated by the temperature of the external environment. Although it seems a simple process, its temperature requirements in the process of production, transportation and storage are still strict. Therefore, it is necessary to fully consider the differences in temperature caused by environmental factors (i.e., region or season), as well as physiological factors (i.e., age).

### Dual-responsive hydrogel dressing

Individual variability may lead the effects of singly-responsive hydrogels to vary greatly in diabetic wounds, whereas dual-responsive hydrogels can compensate for each response to achieve better therapeutic effects. Furthermore, since the environmental state of the wound changes over time, different medications are required at different times. And dual-responsive hydrogel dressings can accomplish spatiotemporal controlled release of drugs to avoid the development of drug resistance or other undesirable side effects.

#### pH/ROS-responsive hydrogel dressing

**Fig. 8:**
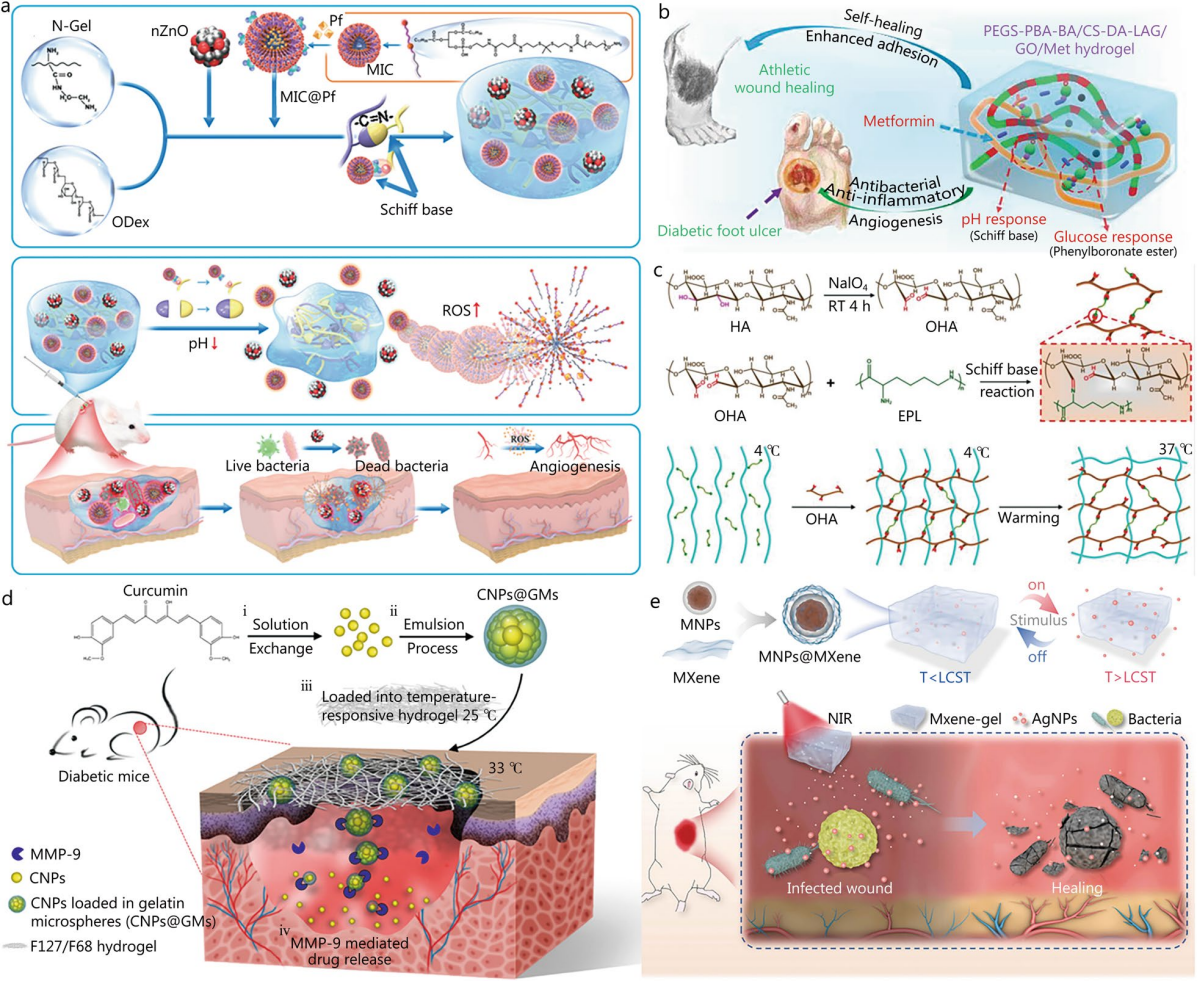
Construction process and structure of representative double-responsive hydrogels.** a** The fabrication of nZnO- and MIC@Pf-Loaded hydrogels and the potential mechanisms of the prepared pH/ROS-responsive hydrogel to facilitate the wound-healing process. Reproduced with permission [89]. Copyright 2022, American Chemical Society. **b** The structure, pH and glucose-responsive mechanism of the PC hydrogel and its application in diabetic foot ulcers and athletic wound healing. Reproduced with permission [26]. Copyright 2022, American Chemical Society. **c** The synthesis of injectable pH/temperature-responsive FHE hydrogel (F127/OHA-EPL) with multifunctional properties. Reproduced with permission [66]. Copyright 2019, Ivyspring International. **d** The preparation and process of a temperature/enzyme-responsive hydrogel with drug release at the wound bed in diabetic mice. Reproduced with permission [70]. Copyright 2018, American Chemical Society. **e** The formation and drug release process of the MXene-based photo/magnetic-responsive hydrogel. Reproduced with permission [109]. Copyright 2021, Wiley‐VCH GmbH. CNP curcumin nanoparticle, CS-DA-LAG dihydrocaffeic acid and L-arginine cografted chitosan, EPL poly-ε-L-lysine, GO graphene oxide, HA hyaluronic acid, LCST lower critical solution temperature, Met metformin, MMP matrix metalloproteinase, MNPs magnetic nanoparticles, MIC micelles, NIR near-infrared, N-Gel ethylenediamine-modified gelatin, nZnO zinc oxide nanoparticles, ODex oxidized dextran, OHA oxidized hyaluronic acid, PBA phenylboronic acid group, PEGS-BA polyethylene glycol-co-poly(glycerol sebacic acid), Pf paeoniflorin, ROS reactive oxygen species, RT room temperature, AgNPs silver nanoparticles

Diabetic wounds tend to induce persistent bacterial infection, resulting in a lower pH, extending the inflammatory period and leading to a further increase in ROS concentration, which increases the difficulty of clinical treatment. pH/ROS-responsive hydrogel dressings enable sequential drug release that can provide a more favorable environment for subsequent drug release and better fit the diabetic wound healing process to improve drug availability and accelerate wound healing. Guo et al*.* [89] designed pH/ROS-responsive hydrogels infused with antimicrobial zinc oxide nanoparticles (nZnO) and paeoniflorin (Pf)-coated micelles. The pH responsiveness of hydrogels is enabled by a dynamic Schiff-base of ethylenediamine-modified gelatin and ODex (Fig. 8a). Gelatin alone has strong hemostatic characteristics, while Pf is an angiogenic compound contained in micelles made by the amphiphilic polymer DSPE-TK-PEG2k-NH2. The mercaptan group contained in thioketal (TK) makes the micelles ROS-responsive. As a result, the hydrogel dressing not only enhances hemostasis, but also allows for the sequential release of antibacterial and angiogenic medicines, considerably accelerating the process of diabetic wound healing in diabetic rats. Antibacterial polypeptide ε-polylysine contains amino groups that create Schiff-base bonds and catechol groups that produce boron ester linkages. Wu et al*.* [83] used phenylboronic acid-grafted oxyglucan and caffeic acid-grafted ε-polylysine (CE) to form hydrogels with two dynamic covalent bonds: Schiff-base and boron ester bonds. The hydrogels were loaded with the anti-inflammatory drug diclofenac sodium (DS) and pH-responsive micelles (MIC@MF) containing the angiogenic drug mangiferin (MF). In the diabetic wound site’s low pH and high ROS environment, DS and CE are released quickly to promote anti-bacterial conditions and limit inflammation, while MF is released slowly and continuously to promote angiogenesis. This temporal- and spatial-specific release of medicines could accelerate the process of wound healing in diabetic rats (SD). Such dual-responsive wound dressings provide a promising platform for the clinical application of sensitive drugs or drugs with specific spatiotemporal action requirements, although the preparation of such dressings may be more rigorous and require a more accurate evaluation of the release of different drugs.

#### pH/glucose-responsive hydrogel dressing

Glucose-responsive hydrogel wound dressings typically generate pH changes and pH value at the wound site significantly affects the function of some drugs and the progress of wound healing. A pH/glucose-responsive hydrogel application can compensate for this side effect of glucose-responsive hydrogel dressings by minimizing the change in pH value at the wound site. Zhao et al*.* [107] reported a pH- and glucose-responsive hydrogel dressing based on benzoic-imine bonds and phenylboronate ester to deliver insulin and fibroblasts for the treatment of diabetic wounds. Insulin is continuously secreted in the low pH and high glucose milieu of diabetic wounds to improve wound healing by activating insulin signaling pathways, while fibroblasts enhance angiogenesis by generating ECM molecules and secreting growth factors. In vivo results showed that wound healing, neovascularization, and collagen deposition were increased in STZ-induced diabetic rats treated with insulin/L929 hydrogel dressings. L-arginine (LAG) is thought to promote insulin secretion, which can provide good support for diabetic wound healing. Metformin (Met) is a first-line medicine in the clinical treatment of type II diabetes that can increase the sensitivity of local cells to insulin, but it has a limited oral bioavailability and a short half-life. Liang et al*.* [26] developed a multi-functional hydrogel dressing, PC/GO/Met, with adhesive antibacterial, antioxidant and conductive properties (Fig. 8b). For example, the Schiff-base bond and phenylborate bond formed between PEGS-PBA-BA/CS-DA-LAG (PC) can respond to pH and glucose at the wound site, allowing the hydrogel to not only achieve specific drug release but also to have good removal ability. Animal experimental results showed that the quality of regenerated skin of type II diabetic SD rats in the PC hydrogel treatment group was better than that in the control group, with abundant granulation tissue, increased collagen deposition, better hair follicle regeneration, and more obvious angiogenesis. pH/glucose-responsive hydrogels not only allow pH and glucose responses to promote each other, but also to compensate for each other, thus accelerating diabetic wound healing. However, the blood glucose levels of diabetic patients fluctuate, and individuals differ greatly. Whether this will affect the drug release behavior of the dressing needs to be fully evaluated before clinical trials.

#### pH/temperature-responsive hydrogel dressing

Hydrogel dressings that are pH/temperature-responsive have demonstrated several advantages in diabetic wound healing, for instance, they rapidly form a gel at the wound site, increasing adhesion and local concentration of drugs, and subsequently eliminate the gel through degradation when pH changes.

PNIPAM has a LCST of 32.0 °C and can spontaneously undergo a phase transition from hydrophilic (soluble) to hydrophobic (insoluble) chains. The LCST is often changed by modifying hydrophilic monomers. Haidari et al*.* [22] prepared a hydrogel with temperature- and pH-responsiveness by cross-linking N-PNIPAM and acrylic acid (AAc) loaded with ultrafine AgNP that could be released according to microenvironmental changes to efficiently suppress bacterial infection at the wound site. The LCST of N-PNIPAM rose to 36.5 °C after co-polymerization with AAc and hydrogel was formed when it was close to human body temperature, while AAc, as a weak acid, induced hydrogel swelling after pH rise and gradually released AgNPs. In vivo experiments indicated that the intelligent hydrogel PNIPAM-PAA-AgNPs could release Ag ions on demand to achieve high antibacterial effect in *S. aureus*-infected rat wounds, which could provide guidance for clinical transformation and treatment of chronic wounds.

Pluronic F127 are amphiphilic block copolymers with a PEG-PPG-PEG structure, and their 20–30% w/w aqueous solutions have the unusual feature of reverse thermal gelation, meaning they are liquid at cold temperatures (4–5 °C) but gel when heated to room temperature [23]. Wang et al*.* [66] synthesized oxide hyaluronic acid (OHA), which forms Schiff-base bonds with poly-ε-L-lysine (EPL), and introduced thermally-responsive F127 to form injectable FHE hydrogels (F127/OHA-EPL) that deliver adipose-derived mesenchymal stem cell (AMSC)-exo in response to weak acidic environments (Fig. 8c). This multi-functional hydrogel with controllable EXO release led to faster granulation tissue formation, and accelerated the wound healing process in mice. The dual response to pH and temperature can better regulate the gel formation and degradation of the dressing. However, skin temperature is easily affected by the external environment and other factors. Thus the temperature-responsive dressing must not only meet the conditions of gel formation but must also not affect the gel degradation process triggered by pH response. This may create more stringent requirements for the preservation and use of such wound dressings, which may limit their clinical application.

#### Temperature/enzyme-responsive hydrogel dressing

Temperature is a key factor in both the formation of thermosensitive hydrogels and the activity of enzymes. Within a certain range, as temperature increases, the gel shrinks to release drugs, while enzyme activity increases, further promoting the rapid release of drugs. Thermosensitive hydrogels can improve the stability of drug delivery and have a good loading capacity for hydrophobic and insoluble drugs. Curcumin is a natural polyphenol with antioxidant and anti-inflammatory properties that contribute to wound healing at all stages. However, since curcumin is a small hydrophobic molecule with poor solubility, excessive quantities of curcumin can cause mitochondrial dysfunction, and it is thus critical to establish an efficient and regulated delivery system. Liu et al*.* [70] encapsulated the curcumin nanoparticles (CNPs) formed by self-assembly of curcumin in gelatin (CNPs@GMs) (Fig. 8d). The gelatin responds to the overexpression of MMP-9 at the wound site by increasing the concentration of curcumin. To achieve the slow and sustained release of the drug, CNPs@GMs is combined with the temperature-sensitive hydrogel F127 to form a dual-response hydrogel dressing. This hydrogel dressing is capable of specific curcumin release at the wound site, enhancing the action of the drug and reducing cell damage to promote the recovery of wound skin structure and function in diabetic mice. However, this kind of hydrogel dressing needs to meet certain mechanical properties and hydrophilicity to ensure that the enzymes at the wound site can enter the gel. Besides temperature, enzyme activity is also known to be affected by pH and this may affect the clinical efficacy of the dressing.

#### Photo/magnetic-responsive hydrogel dressing

In addition to stimulation of the microenvironment at the wound site, several external stimuli such as light, magnetic fields and electric fields can also be applied to the smart drug delivery system. Magnetic field and near infrared light are the first choices for deep wound treatment due to their strong tissue penetration and high sensitivity. Yang et al*.* [109] selected MXene-based hydrogel to wrap Fe_3_O_4_@SiO_2_ magnetic nanoparticles (MNPs@MXene) with magnetic and NIR thermal conversion, and further integrated them into a PNIPAM-alginate dual network hydrogel (NIPAM-Alg) (Fig. 8e). To increase the antibacterial characteristics of the hydrogel system, AgNPs were added. MNPs create heat when exposed to NIR light or repeatedly stimulated by an external alternating magnetic field, causing the hydrogel to quickly warm up, shrink, and release medicines. The experimental results of AgNPs release show that applying external stimulation to MXene-based hydrogels can achieve precise and controllable drug release. This NIR-induced intelligent drug release hydrogel has been demonstrated to show satisfactory performance in deep infected wound treatment. The wound environment is affected by various factors and thus dressings must be highly sensitive to change; external regulation may alleviate this concern. However, compared with environmental stimulation, while external stimulation can regulate drug levels, it requires accurate and timely assessments of the wound-healing process. Therefore, it may be more suitable for use under the supervision of relevant professionals, rather than for daily use by patients.

## Conclusions and perspectives

The complex diabetic wound microenvironment (including hyperglycemia, low pH, high ROS, overexpressed enzymes, and mal-regulated growth and inflammatory factor levels) leads to long-term delays of the wound healing process at the inflammatory stage and increased risk of wound infection, posing a challenge for the clinical treatment of diabetic wounds. There are various therapeutic strategies for diabetic wounds, and wound dressing is an important component. However, existing medical hydrogel wound dressings have static effects that may cause maceration in wounds that take a long time to heal. Their application for anti-infection use is also controversial. If combined with antibiotics and other drugs, this may inhibit infection in diabetic wounds but the drug release and degradation rate cannot be accurately regulated based on environmental changes.

In recent years, stimulus-responsive multifunctional hydrogel wound dressings have emerged that respond to changes in pH, glucose, ROS, enzymes, or temperature at the wound site, achieving controlled and sequential drug release. In addition to delivering medicine, responsive-hydrogel wound dressings can improve the harsh environment of diabetic wounds. Thereby, these wound dressings are able to accelerate wound healing and show great potential in clinical diabetes wound treatment. Further, the actual treatment cost of smart responsive-hydrogel wound dressings may be lower than existing medical gel dressings or traditional dressings when treatment time, dressing frequency, recurrence rate and other aspects are considered. However, smart responsive-hydrogel wound dressings have not yet reached the clinical stage, and this may be because some urgent problems remain to be solved.

Firstly, as a drug delivery carrier, responsive hydrogels’ intelligent drug release behavior might be affected by the pH value, glucose level, or enzyme activity in wounding environment. Therefore, it is necessary to strictly evaluate the effects of pH change, glucose level, ROS level, temperature change or enzyme activity on drug release behavior, especially for smart hydrogels with multiple responses. In addition, smart responsive wound dressings need to be stable before they can be applied. However, oxidizing substances, enzymes, temperature changes and other environmental factors in the process of production, storage and transportation may also affect the behavior of gel formation, drug loading and drug release of hydrogel wound dressings. This also puts forward higher requirements for the production, transportation and storage of wound dressings.

Secondly, attention should be paid to the animal models used in the study before proceeding to clinical trials of responsive hydrogels. Common animal models of diabetic wounds (mice, rats, and pigs) each have their advantages and disadvantages in preclinical studies. For example, mouse diabetic wound models are low-cost, easy to operate and maintain. While rats are larger than mice, and multiple reagents can be tested per animal. Porcine skin is more similar to human skin in terms of anatomy and physiology [29], but it is more expensive and difficult to manipulate. Although no animal model can reproduce the process and changes of diabetic wound healing, the success rate of clinical transformation will be higher if the animal model is selected to better fit the human model. Once the glucose level, pH value, enzyme expression level and other factors in the chronic wound environment of diabetic patients are significantly different from the experimental animal diabetic wound model, it might lead to large errors in the gel formation time, drug release behavior and degradation time of responsive wound dressings in clinical trials, thus leading to unsatisfactory treatment results. Furthermore, the animal experiment period of preclinical studies (usually 2–3 weeks) is shorter than the actual development of wounds in diabetic patients, such as DFU clinical healing cycle of more than 4 weeks, and prone to recurrence. Therefore, the long-term effect of smart hydrogel on diabetic wound remodeling needs to be further studied. In conclusion, animal models similar to human skin structure should be selected as far as possible in preclinical studies, and the treatment and observation period can be prolonged to provide more confidence for clinical trials.

Last but not least, since wound healing is a dynamic process, the glucose level, pH, ROS and other factors at the wound site also fluctuate which might affect the wound healing process. For example, low concentration ROS have antibacterial and angiogenic effects, while high concentration ROS can harm cells and delay wound healing. Therefore, clinical use of responsive-hydrogel dressings requires timely and accurate assessment of the wound environment changes in diabetic patients. Furthermore, on the one hand, there might be individual wound environment differences in diabetic patients while on the other hand, the wound environment of patients might also change at different stages of development. Therefore, it is necessary to establish reliable clinical monitoring indicators to guide clinical applications and to select suitable responsive wound dressings according to patients’ wound conditions.

Although clinical measuring devices such as infrared thermometers, pH strips, and glucose monitors can be used to measure temperature, pH, and glucose levels at the wound site, respectively. The use of these instruments is independent of wound dressings and cannot track environmental changes such as pH and glucose levels in the wound in real time. At present, many studies have designed wound dressings that integrate diagnosis and treatment, which can promote wound healing while monitoring pH value, glucose level or wound infection. It is believed that this will be the development direction of smart hydrogels in the future.

However, no one product is perfect for all diabetic wounds. Therefore, the construction of a systematic monitoring system and an external control system is necessary to facilitate more individualized wound care for diabetes patients. The preparation, storage and use of this smart responsive hydrogel might seem complex, but for complex chronic diabetic wounds such as DFU, personalized treatment can often achieve more efficacy. As the population ages and the number of people with diabetes increases, the promotion of smart responsive hydrogel dressings may alleviate the economic pressure of chronic diabetic wound care in general, if the probability of wound recurrence, the risk of infection and the cost of dressing changes are taken into account. More importantly, there is a growing focus on wound management and a wider market for smart responsive hydrogels that combine innovation and quality. Therefore, we are confident that with the gradual solution of the above problems, responsive hydrogel wound dressings will gradually reach the clinic and become the first choice of wound care materials for diabetic patients and medical personnel.

## Data Availability

Not applicable.

## Abbreviations

AAc: Acrylic acid
ADH: Adipic acid dihydrazide
ADSC-exo: Adipose-derived stem cell exosomes
AFPBA-ins: 4-(2-Acrylamide ethyl aminoformyl)-3-fluorobenzene boric acid with insulin
AGEs: Advanced glycosylation end products
AgNPs: Silver nanoparticles
AMSC: Adipose-derived mesenchymal stem cell
AMP DP7: Antimicrobial peptide DP7
ASC: Allogenic mesenchymal stem cells
BG: Bioglass
CNP: Curcumin nanoparticle
Con A: Concanavalin A
COL-1: Collagen-1
CS-DA-LAG: Dihydrocaffeic acid and L-arginine cografted chitosan
DFU: Diabetic foot ulcer
DFO: Deferoxamine
DG@Gel: Glucose-responsive multifunctional metal–organic drug-loaded hydrogel
DS: Diclofenac sodium
ECM: Extracellular matrix
EPL: Poly-ε-L-lysine
EXO: Exosomes
FHE hydrogels: F127/OHA-EPL
G-insulin: Gluconic insulin
GM-CSF: Granulocyte-macrophage colony-stimulating factor
GO: Graphene oxide
GOX: Glucose oxidase
HA-ALD: Hyaluronic acid–aldehyde
HTCC: Chitosan quaternary ammonium salt
HNTs: Halloysite clay nanotubes
ICG: Indocyanine green
IDA: 4,5-Imidazoledicarboxylic acid
IL-6: Interleukin-6
IL-1β: Interleukin-1β
LAG: L-arginine
LCST: Lower critical solution temperature
LED: Light emitting diode
MAL: Maleimide group
MDR: Multiple drug resistance
Met: Metformin
MF: Mangiferin
MIC: Micelles
MMP: Matrix metalloproteinase
MMP(W)x: The substrate peptides of MMP
MNPs: Magnetic nanoparticles
MNPs@MXene: Fe_3_O_4_@SiO_2_ magnetic nanoparticles
MRSA: Methicillin-resistant *Staphylococcus aureus*
NCT: National Clinical Trial
N-chitosan: N-carboxylethyl chitosan
N-Gel: Ethylenediamine-modified gelatin
NIR: Near-infrared
NPs: Nanoparticles
N-PNIPAM: N-isopropylacrylamide
nZnO: Zinc oxide nanoparticles
OCMC: Oxidized carboxymethyl cellulose
ODex: Oxidized dextran
OD-DA: Oxidized dextran-dopamine
OHA: Oxidized hyaluronic acid
OSA: Oxidized sodium alginate
PBA: Phenylboronic acid group
PC: PEGS-PBA-BA/CS-DA-LAG
PDGF: Platelet-derived growth factor
PEI: Polyethyleneimine
PEG: Polyethylene glycol
PEG-DA: Poly (ethylene glycol) diacrylate
PEGS-BA: Polyethylene glycol-co-poly(glycerol sebacic acid)
Pf: Paeoniflorin
PPBA: Phenylboric acid-modified polyphosphazene
PVA: Polyvinyl alcohol
QCS: Quaternized chitosan
ROS: Reactive oxygen species
RT: Room temperature
*S. aureus*: *Staphylococcus aureus*
SA: Sodium alginate
SF: Silk fibroin
STZ: Streptozocin
TA: Tannic acid
TK: Thioketal
TNF-α: Tumor necrosis factor-α
TPA: N1-(4-boronobenzyl)-N3-(4-boronophenyl)-N1, N1, N3, N3-tetramethylpropane-1, 3-diaminium
UCST: Upper critical solution temperature
UV: Ultraviolet
VEGF: Vascular endothelial growth factor
